# The Story of the Insane from Year to Year

**Published:** 1901-11-09

**Authors:** 


### THE STORY OF THE INSANE FROM YEAR TO YEAR.

(Thirteenth Series.)

### MONTROSE ROYAL ASYLUM.

This is a venerable institution, for nearly 120 years have passed away since it was opened. At the beginning of their statistical year (May 15th, 1899) it contained 670 patients, and on the corresponding date of 1900 there remained under treatment 652; of these 549 were paupers, and 103 were private cases. There were in all 138 first admissions, 26 re-admissions, 68 recoveries, 55 discharged relieved, and 59 deaths. The average number resident was 659, and the total number of persons under treatment during the year was 832. Calculated on the admissions the percentage of recoveries stands at 41·35, and that of the deaths calculated on the average number resident at 8·95. Of the deaths 19 were due to cerebral and spinal diseases, 6 to consumption, 5 to pneumonia, and 1 to pleurisy. The factors of insanity usually called moral causes account for 17 of the year's admissions; intemperance in drink accounts for 22, hereditary influence for 18, and 41 are put down as of unknown cause. Such a high proportion of "unknowns" vitiates the whole table, and we think that inquiry by the parish officers previous to admission and investigation by the asylum officers after admission might have removed many from this class. During the last decade or so the cause "influenza" has often appeared in the statistics of all our asylums; and that it is often used as a sort of scapegoat there can be no doubt. Dr. Havelock very well expresses this in the following sentences: "There has been a tendency," he says, "in some quarters to exaggerate the influence of this disease as a factor in the production of mental disturbance. Perhaps it has been overlooked that there is a natural inclination to date back all mental troubles to some debilitating bodily disease, and as few people escape influenza nowadays—or at any rate what is loosely termed influenza—it is stated as the cause in a great many doubtful cases in preference to habits and inherited tendencies which are not so readily divulged." The asylum is full, and a detached block for male patients is being erected. The commissioners say that "marked ability is shown both in the general and medical management of the institution;" and the committee "record their satisfaction with the services of their physician superintendent and his staff.

